# Treatment of Diphtheria by the French School

**Published:** 1878-09

**Authors:** 


Treatment of Diphtheria by the French School.—Diphtheria is very prevalent just now in the French capital, and naturally occupies the attention of the most eminent practitioners in Paris. Hitherto the method by "substitution," or the conversion of the specific into simple inflammation, has been the one usually employed, particularly by Bretonneau and his illustrious successor,

Trousseau. The latter, however, was fully acquainted with the specific nature of the diphtheria poison, yet continued the practice which experience had shown to be utterly futile. It has been remarked, on the other hand, that the failure of local remedies depends on the circumstances that those employed are not addressed to the specific elements of the disease. M. Revillent's experience leads him to conclude that we possess such a remedy in lemon juice when applied to the false membranes, and also administered internally in large doses. It would truly seem to be a specific, as the author assures us that he has not lost a single case in which this method has been fully and fairly carried out.—*London Med. Examiner.*

